# Commercially Sexually Exploited Adolescent Girls: The Association Between Externalizing Disorders and Parental Incarceration with Suicide Attempts

**DOI:** 10.1007/s10578-024-01730-1

**Published:** 2024-06-27

**Authors:** Sara J. Schiff, Jocelyn Meza, Eraka Bath, Steve S. Lee

**Affiliations:** 1 Department of Psychology, University of California Los Angeles, 502 Portola Plaza, Franz Hall, Los Angeles, CA 90095-1563 USA; 2 Department of Psychiatry and Biobehavioral Sciences, David Geffen School of Medicine at UCLA, Los Angeles, CA USA

**Keywords:** Commercial sexual exploitation, Externalizing problems, Parental incarceration, Suicide, Adolescent girls

## Abstract

Commercial sexual exploitation (CSE) is a significant public health concern disproportionately affecting ethnoracially minoritized girls. Despite strong associations of CSE with suicide attempts, little is known about correlates of suicide among girls with CSE histories. Elevated rates of externalizing disorders (i.e., attention-deficit/hyperactivity disorder [ADHD], disruptive behavior disorders [DBD], conduct disorder [CD]) were observed among CSE youth, particularly in ethnoracially minoritized samples. Youth with CSE histories are frequently affected by parental incarceration, which is correlated with risk for suicide attempts. We tested cross-sectional simultaneous associations of externalizing disorders and parental incarceration with number of suicide attempts among 360 ethnoracially diverse girls affected by CSE (Mean age = 18.86). ADHD, DBD, and maternal incarceration were positively associated with number of suicide attempts. Findings implicate clinical/familial correlates of suicide attempts in this marginalized group, suggesting early suicide prevention efforts may improve traction on this problem by focusing on individual and family level factors.

## Introduction

Commercial sexual exploitation (CSE) is defined as the exchange of sexual acts for something of value (e.g., money, shelter, goods, acts of service) [1]; when CSE involves minors who are trafficked and receive compensation, it is recognized as a form of child maltreatment [2]. Critically, adolescents impacted by CSE disproportionately experience multiple risk factors across clinical, environmental, and systemic domains, including acute unmet health needs [3]. Limited self-disclosure by affected adolescents, difficulty identifying victims, and the lack of a centralized reporting database complicate precise prevalence estimates of adolescents impacted by CSE; however, between 2008 and 2010, 82% of all suspected trafficking incidents in the United States were categorized as sex trafficking, including over 1,000 cases of child sex trafficking [4]. Federal and state legislation (e.g., in California) established that individuals with a CSE history under the age of 18 should be considered victims of severe trafficking, effectively diverting these individuals from the juvenile legal system to the child welfare system [1]. Coupled with replicated evidence that ethnoracially minoritized adolescents are particularly vulnerable to CSE, there is an urgent need to identify risk and protective factors to accelerate intervention development for this subgroup of the population [5]. Indeed, data from the Bureau of Justice Statistics estimated that nearly 95% of all sex trafficking victims were female, 54% were 17 years old or younger, a third were Black/African American, and about one fifth were Hispanic/Latinx. These data further underscore how ethnoracially minoritized girls are particularly overrepresented in this population [4]. Considering the intersectionality of sex trafficking victims (i.e., female, ethnoracially minoritized), future research must carefully consider these specific marginalized groups to develop effective intervention strategies and prevention policies.

Adolescents affected by CSE are structurally vulnerable to experiencing a range of adverse medical and behavioral health problems and frequently suffer from significant gaps in obtaining adequate health services. Specifically, suicidality has been implicated with many of the individual risk factors associated with CSE. Indeed, suicide is the second leading cause of death for youth 10–14 years old and the third leading cause of death for individuals 15–24 years old [6]. These distressing trends are amplified among ethnoracially minoritized youth (i.e., Black and Latinx) for whom rates of suicide attempts and death by suicide are increasing more precipitously than non-Hispanic White youth [7]. For example, rates of suicide attempts are highest among Black and Latinx adolescents (11.8% and 8.9%, respectively), compared to their non-Hispanic White counterparts [8]. In addition, in community samples, recent trends suggest a steep rise in suicide rates among Black youth specifically [9]. Thus, there is an urgent need to identify risk factors for suicide attempts among structurally vulnerable (i.e., adolescents impacted by CSE) groups who are disproportionately Black and Latinx; this evidence is necessary to intervene and prevent this severe outcome among this ethnoracially minoritized population.

Layered on top of the disproportionate risk of suicide in Black and Latinx adolescents are gender disparities regarding the specific suicidal outcome of suicide attempts. Indeed, there exists longstanding evidence that girls are twice as likely as boys to attempt suicide [8]. In particular, a recent systematic review observed girls had a higher pooled risk of suicide attempts compared to boys, with 12–24 year old girls having both a higher lifetime prevalence and 12-month incidence of suicide attempts [10]. Even more, race-ethnicity and gender interact to produce compounding risk for suicidal thoughts and behaviors, which are robust predictors of suicide attempts [11]: Black adolescent girls have the highest rates of suicidal thoughts and behaviors overall and demonstrated the sharpest rise in suicide rates over time compared to other racial-ethnic and gender groups [12]. In addition, in 2019, 15.2% of Black and 11.9% of Latinx female high school students reported a suicide attempt, compared to 9.4% of non-Latinx White female students [8]. Whereas rates of suicide attempts have been stable or decreased for many racial-ethnic groups, rates have increased significantly among Black girls in particular [13]. These data highlight the potency of gender and race-ethnicity-based risk factors for suicide attempts, and by extension the amplified risk for subsequent death from suicide. This is especially salient among subgroups (i.e., ethnoracially minoritized groups, female identity) who are structurally marginalized populations at risk for poor mental and physical outcomes.

Number of previous suicide attempts is a highly consequential outcome considering previous suicide attempts are one of the strongest predictors of future suicidal behavior, including suicidal ideation, additional suicide attempts, and death by suicide [14–18]. Indeed, suicide attempts are an even more lethal risk factor for completed suicide than originally thought after potential biases were accounted for (i.e., disregard of previous attempts, overlooking first-attempt deaths) [19]. Although most previous research has examined all suicide attempters in one single category (i.e., dichotomous yes or no), evidence indicates that youth who make multiple suicide attempts may represent a distinct profile from those who report a single attempt, such that multiple suicide attempters have more severe reported psychopathology [20]. More importantly, multiple attempts are strong indicators of overall suicidal severity (i.e., more frequent wish to die during attempt) and are predictive of later suicide attempts [21]. As such, considering the number of suicide attempts an individual has made in their lifetime is necessary to adequately capture the severity of suicide risk; however, this continuous outcome is infrequently used in the evidence base.

Critically, adolescents with a history of CSE are at an increased risk for suicidal thoughts and behaviors. Specifically, adolescents with a CSE history exhibit more frequent suicidal thoughts compared to adolescents without a CSE history [22]. In addition, a California study of adolescents affected by CSE estimated that 35% of youth engaged in moderate to severe self-harm, which is nearly twice as prevalent relative to national trends; 12% also endorsed a suicidal gesture, attempt, or plan to attempt suicide in the previous 30 days [23, 24]. Persuasive meta-analytic evidence also suggests that early life sexual abuse is cross-sectionally and prospectively associated with a nearly twofold increase in risk for suicide attempts [25]; similar results were found in an earlier meta-analysis, even with control of multiple covariates (e.g., genetic factors, early family environment) [26]. Given that CSE is considered a type of childhood sexual abuse, there is convincing evidence that suicidality amongst CSE populations is a significant concern worthy of investigation. However, despite this acute risk for suicidality (i.e., suicide attempts) among adolescents affected by CSE and their vulnerability to suicidal outcomes (i.e., self-injury, death), these patterns await rigorous characterization specifically among ethnoracially minoritized girls. In other words, despite the prevalence and significance of suicidality among CSE-impacted adolescents, relatively few risk factors have been identified specifically in ethnoracially minoritized girls, who constitute the vast majority of adolescents with histories of CSE. Thus, the present research seeks to identify specific correlates of number of suicide attempts, an acute risk factor for death by suicide, among ethnoracially minoritized girls impacted by CSE.

In addition to elevated suicide risk, systems-involved adolescents exhibit elevated rates of externalizing disorders [27], particularly adolescents affected by CSE [23, 28, 29]. For example, in an adjudicated delinquent sample, CSE-impacted youth were nearly four times more likely than non-CSE-impacted youth to receive a clinical “hostility” diagnosis (i.e., composite of externalizing behaviors such as irritability, urges to break things, uncontrollable temper, outbursts, and argumentativeness) [30, 31]. Similarly, among confirmed and at-risk 12–18 year-old girls with CSE histories, significant attentional and related conduct problems (e.g., aggression) were consistently observed [32]. In a subset of the present study’s sample, 40% and 32% of adolescents affected by CSE had a lifetime diagnosis of a disruptive behavior disorder (DBD; including oppositional defiant disorder) and attention-deficit/hyperactivity disorder (ADHD), respectively [27]. Crucially, however, adolescents affected by CSE with significant trauma histories can present clinically with problems that mirror common externalizing behaviors (e.g., irritability, poor concentration). Indeed, qualitative narratives have highlighted that traumatic stress experienced by CSE-impacted individuals is often persistent and intractable [33]. Further, nearly 80% of women and girls with histories of CSE are diagnosed with PTSD [34]. Given that traumatic stress shares overlapping features with externalizing problems, precise inferences about the association of ADHD and other externalizing problems (i.e., DBD, CD) with suicide attempts requires careful consideration of co-occurring trauma histories.

Despite ADHD and related externalizing problems being risk factors for suicide in girls in the general population across multiple studies and being independently prevalent among adolescents with CSE history, their specific associations with suicide risk among girls with CSE histories have not been investigated. Indeed, in a 10-year prospective study, ethnically diverse adolescent girls with ADHD exhibited elevated rates of suicide attempts and non-suicidal self-injury relative to comparison youth [35]. In another study with the same sample, 20% and 5.8% of ADHD and matched control girls, respectively, had a history of suicide attempts; childhood ADHD symptoms and early adverse childhood experiences also robustly predicted suicide attempts 16 years later [36]. Even more, in a large Canadian sample, 25% of women diagnosed with ADHD had a lifetime history of a suicide attempt [37] and a recent meta-analysis highlighted that ADHD was strongly and positively associated with suicidal spectrum behaviors, including suicide attempts [38]. Despite the replicated evidence that ADHD is a risk factor for suicidality in girls, it is unclear if/how ADHD relates to suicidality among ethnoracially diverse girls with CSE histories, specifically. Externalizing problems more generally (e.g., CD, delinquency) were also cross-sectionally and longitudinally associated with higher risk for suicidality [39–41]. Among a mixed-sex adult population, externalizing problems (i.e., impulsivity, deviant behaviors) were significantly associated with history of suicide attempts [42]. However, again, this has not been prosecuted among high risk, adolescent populations. To provide much needed empirical evidence, the present study investigated the independent associations of externalizing disorders including ADHD, DBD (including oppositional defiant disorder), and CD with number of suicide attempts among ethnoracially diverse adolescent girls with histories of CSE.

In addition to the potential co-occurring risk factors outlined above, adolescents affected by CSE often experience family instability and parental antisocial behavior (e.g., substance abuse, criminality; [43, 44]). In a study using the present sample, 5% of girls with CSE histories had mothers who had been incarcerated and 14% had fathers who had been incarcerated, key indicators of family instability [45]. Indeed, parental incarceration was associated with making a suicide plan and suicidal behavior as well as elevated ADHD and CD [46–48], highlighting the need to consider parental incarceration in emerging models of suicide attempts. Further, among African American/Black adolescents, maternal incarceration, but *not* paternal incarceration, positively predicted adolescents creating a suicide plan [49]. Thus, maternal vs. paternal incarceration may be differentially associated with adolescent suicidality.

### Current Study

The current study aimed to address crucial knowledge gaps that prevent innovations in intervention development and implementation. We intensively characterized a high-risk population of 360 ethnoracially diverse adolescent girls with CSE histories who were involved in the specialty Succeeding Through Achievement and Resilience (STAR) Court in Los Angeles (LA). We examined the concurrent, simultaneous associations of externalizing disorders (i.e., ADHD, DBD, CD) and parental incarceration with number of suicide attempts made in participants’ lifetimes; as noted above, given the density of risk factors in this population, we adjusted for age, race-ethnicity, and traumatic stress. We hypothesized that ADHD, DBD, and CD would each be uniquely and positively associated with number of suicide attempts over and above key covariates. We also hypothesized that parental incarceration would be associated with more suicide attempts and that maternal, but not paternal, incarceration would similarly be positively associated with number of suicide attempts.

## Methods

### Setting

Exhaustive case file reviews were conducted for all youth involved in the STAR Court in LA County from 2012 to 2016. The STAR Court is an innovative specialty court program designed for youth with histories of CSE. The Federal Bureau of Investigations (FBI) and the US Department of Justice recognized LA County, in particular, as a high intensity area for youth affected by CSE [50, 51]. Since its creation in 2012, the STAR Court has partnered with local group homes and social service agencies and provided specialized services to adolescents with CSE histories currently on probation for various criminal charges (e.g., “prostitution”). In particular, the STAR court provides ongoing assessment of psychosocial needs and facilitates referral and linkage to rehabilitative and health-related services to ensure and promote safety, education, mental health, and medical care. STAR Court team members include personnel trained in trauma-focused, survivor-centered care and they utilize various harm reduction strategies targeted specifically to CSE-involved adolescents. Inter-agency collaboration between LA County’s Public Defender’s Office, Department of Children and Family Services, Department of Public Health, Department of Probation, Department of Mental Health, and community-based service providers is critical to the success of this voluntary program.

### Data Collection

As noted above, a retrospective and exhaustive case file review was conducted on adolescents involved in LA County’s STAR Court from January 2012 to December 2016. Data from STAR Court files are sourced from the LA County Juvenile Court System, the LA County Department of Children and Family Services, the LA County Probation Department, educational and mental health records, and additional information from group homes and other social service agencies. Data extraction from the paper case files began in February 2015 and were uploaded into RedCap, a secure online database in compliance with the Health Insurance Portability and Accountability Act of 1996. Two research assistants, under the supervision of a postdoctoral research scholar with public health training, examined and coded all case files. Inter-rater reliability was assessed for the first 25 case files on two occasions during the early coding process and when coding discrepancies were found, the team discussed coding decisions until consensus was reached. In addition, all case files were reviewed at the courthouse so that court personnel were available to consult on any questions about case files (see [52] for additional detail on data collection procedures). All study procedures were approved by the UCLA Institutional Review Board and the LA County Superior Court.

### Sample

Eligibility for STAR Court required that adolescents be post-adjudication and must either have disclosed a history of CSE, have been a suspected victim of CSE, or have been considered high risk for CSE. In addition, whereas U.S. citizenship is not required for participation, adolescents must be English-speaking and at least 12 years old. STAR Court participation is voluntary for adolescents during their probation period. Data on 364 case files (360 cisgender females) were reviewed at the time of data collection. Considering the vast majority of cisgender females in the sample and the inability to meaningfully evaluate potential gender/sex differences, analyses excluded case files for non-cisgender females (*n* = 2 cisgender males, *n* = 2 transgender females).

### Variables

Demographic factors in the analyses included age at end of data collection (December 2016), race-ethnicity (i.e., Black/African American, Hispanic/Latinx, White/Other), and dichotomous endorsement of traumatic stress prior to STAR Court involvement (0 = no, 1 = yes). Participants received an endorsement of traumatic stress if traumatic stress was identified in any of the following court records: 730 court-ordered mental health evaluations (completed by a psychologist or psychiatrist), Individualized Education Plan (IEP) school records, and/or behavioral health records. Hypothesized predictor variables consisted of dichotomous diagnostic designations of adolescent ADHD, DBD (including oppositional defiant disorder), and CD prior to STAR Court involvement. Diagnostic status was coded as 0 (no) or 1 (yes) if these diagnoses were identified in any of the following court records: 730 court-ordered mental health evaluations (completed by a psychologist or psychiatrist), IEP school records, and/or behavioral health records. In addition, two separate dichotomous (i.e., yes vs. no) variables for current maternal and paternal incarceration status were combined to create a single parental incarceration variable if either parent was currently incarcerated (0 = no parent currently incarcerated, 1 = at least one parent currently incarcerated). Additional analyses utilized separate maternal incarceration (0 = mother not currently incarcerated, 1 = mother currently incarcerated) and paternal incarceration (0 = father not currently incarcerated, 1 = father currently incarcerated) variables.

Lifetime endorsements of suicide attempts prior to court entry were coded from all data files and court reports available (i.e., 730 court-ordered mental health evaluations, IEP school records, and/or behavioral health records). The dependent variable consisted of two items which were combined to estimate the total number of suicide attempts. One item indicated a negative (= 0) or positive lifetime history of a suicide attempt (= 1); for those who endorsed a lifetime history, a second item reflected the number of suicide attempts made (range 1–15). An overall count variable ranged from 0 (no lifetime suicide attempt) to 15 lifetime suicide attempts. This approach, based on a count of the total number of suicide attempts, better captures the severity of suicidal behavior. For example, multiple suicide attempts is more strongly associated with mortality vs. a single attempt given that most individuals who make a single suicide attempt do not make another attempt later in life [53]. Moreover, among individuals with multiple suicide attempts, the most recent attempt had greater lethality compared to individuals with a single suicide attempt [54]. Thus, modeling risk factors for the highest risk for suicidality based on the severity of suicide attempts is strongly justified.

### Analyses

SPSS 27 was used to analyze data, including initial demographic characteristics and chi-square tests of mean differences for key variables across a dichotomous suicide attempt outcome. Negative binomial logistic regressions modeled the number of suicide attempts from ADHD, DBD, CD, and parental incarceration (i.e., maternal or paternal) controlling for age, race-ethnicity, and traumatic stress. To discern the potential incremental contribution of parental incarceration, additional parallel analyses tested maternal and paternal incarceration as separate predictors rather than the combined parental incarceration variable.

## Results

Demographic information of the 360 cisgender female participants is described in Table 1. The average age of the sample at the end of the study data collection (December 2016) was 18.86 years (*SD* = 1.97, *range* 9.04), 70.3% and 23.1% of the sample identified as Black/African American and Hispanic/Latinx, respectively. 14.7% of CSE adolescents endorsed at least one suicide attempt; the average number of suicide attempts for those who have an attempt history was 2.0. Additional descriptive data on key study variables appears in Table 1. The chi square tests of mean difference for key study variables appears in Table 2; individuals with a suicide attempt history endorsed experiencing past traumatic stress significantly more often those without a suicide attempt history (χ^2^ = 5.40, *p* = 0.02).

**Table 1:** Descriptive statistics of demographics and key variables

	M (SD) or % of Sample	Range
Age	18.86 (1.97)	9.04
Race/Ethnicity		
Black/African American	70.3	–
Hispanic/Latinx	23.1	–
Other (i.e., American Indian, Asian American, Hawaiian Native, White/Caucasian)	6.7	–
Parent incarceration^a^		
At least one parent currently incarcerated	18.1	–
Mother currently incarcerated	5.3	–
Father currently incarcerated	14.7	–
Traumatic Stress endorsement	19.4	–
ADHD endorsement	18.9	–
DBD endorsement	24.7	–
CD endorsement	6.1	–
Ever attempted suicide	14.7	–
Number of suicide attempts for those who have attempt history	2.0 (2.2)	14

*ADHD* attention deficit hyperactivity disorder, *DBD* disruptive behavior disorder (including oppositional defiant disorder), *CD* conduct disorder

^a^Percentages do not total to 100% due to missing data

**Table 2:** Chi-square test of mean differences comparing key variables across a dichotomous suicide attempt history outcome

	With suicide attempt history (*n* = 51) (%)	Without suicide attempt history (*n* = 309) (%)	Chi square test of mean difference	*P* value
ADHD endorsement	21.6	18.4	0.28	0.60
DBD endorsement	25.5	24.6	0.02	0.89
CD endorsement	5.9	6.1	0.01	0.94
Parental Incarceration	22.9	18.2	0.59	0.44
Traumatic Stress endorsement	31.4	17.5	5.40	0.02*

*ADHD* attention deficit hyperactivity disorder, *DBD* disruptive behavior disorder (including oppositional defiant disorder), *CD* conduct disorder

*Association is significant at the 0.05 level

**Fig. 1:**
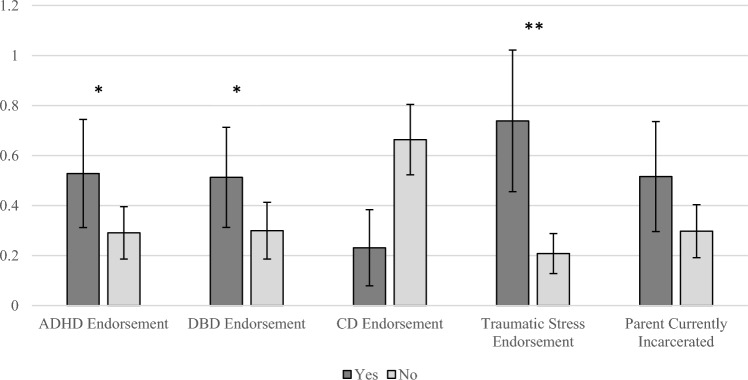
Estimated marginal means of the association of suicide attempts with ADHD, DBD, CD, and parental incarceration. *ADHD* attention deficit hyperactivity disorder *DBD* disruptive behavior disorder (including oppositional defiant disorder), *CD* conduct disorder. **Difference of means is significant at the 0.01 level; *Difference of means is significant at the 0.05 level

We employed negative binomial logistic regression consisting of dichotomous ADHD, DBD, CD, and parental incarceration endorsement as independent predictors of number of suicide attempts, controlling for age, race-ethnicity, and endorsement of traumatic stress (see Table 3). ADHD (*b* = 0.60, *SE* = 0.28, *p* = 0.03) and DBD (*b* = 0.54, *SE* = 0.27, *p* = 0.048) were positively associated with number of suicide attempts. Estimated marginal means suggested that adolescents with a lifetime history of ADHD were expected to have made an average of 0.53 (*SE* = 0.22) suicide attempts whereas non-ADHD adolescents were predicted to make on average 0.29 attempts (*SE* = 0.10; Cohen’s *d* = 0.14; See Fig. 1). For adolescents with a lifetime history of DBD, they were predicted to make 0.51 attempts on average (*SE* = 0.20) whereas adolescents without DBD were predicted to make 0.30 attempts on average (*SE* = 0.11; Cohen’s *d* = 0.12). Traumatic stress and race-ethnicity were also associated with the number of suicide attempts such that individuals with traumatic stress made significantly more suicide attempts than those without traumatic stress (see Table 3). In addition, Hispanic/Latinx individuals were expected to make significantly more attempts than Black/African American individuals. CD endorsement, parental incarceration, and age were not significantly associated with number of suicide attempts made.

**Table 3:** Association of suicide attempts with ADHD, DBD, CD, and 1) parental incarceration, 2) maternal incarceration, and 3) paternal incarceration

Predictors	*b*	*SE*	*p*
Model 1			
ADHD endorsement	0.60	0.28	0.03*
DBD endorsement	0.54	0.27	0.048*
CD endorsement	−1.05	0.66	0.11
Parental Incarceration	0.55	0.31	0.08
Traumatic Stress	1.27	0.27	< 0.001*
Race/Ethnicity			
Black/African American vs Latinx/Hispanic	0.95	0.27	0.001*
Black/African American vs White/Other	0.59	0.46	0.20
Age	0.06	0.07	0.41
Model 2			
ADHD endorsement	0.88	0.30	0.003*
DBD endorsement	0.75	0.30	0.01*
CD endorsement	−1.29	0.80	0.11
Maternal Incarceration	1.25	0.48	0.01*
Traumatic Stress	1.20	0.30	< 0.001*
Race/Ethnicity			
Black/African American vs Latinx/Hispanic	1.01	0.31	0.001*
Black/African American vs White/Other	0.87	0.49	0.07
Age	0.07	0.08	0.35
Model 3			
ADHD endorsement	0.40	0.31	0.19
DBD endorsement	0.69	0.29	0.02*
CD endorsement	−1.15	0.68	0.09
Paternal Incarceration	0.15	0.36	0.67
Traumatic Stress	1.52	0.29	< 0.001*
Race/Ethnicity			
Black/African American vs Latinx/Hispanic	1.09	0.29	< 0.001*
Black/African American vs White/Other	0.48	0.51	0.36
Age	0.08	0.08	0.29

*ADHD* attention deficit hyperactivity disorder, *DBD* disruptive behavior disorder (including oppositional defiant disorder), *CD* conduct disorder

*Association is significant at the 0.05 level

A parallel negative binomial logistic regression model used the same variables, but with maternal incarceration instead of combined parental incarceration as a predictor. ADHD (*b* = 0.88, *SE* = 0.30, *p* = 0.003) and DBD (*b* = 0.75, *SE* = 0.30, *p* = 0.01) were again associated with number of suicide attempts: youth with ADHD and DBD made more suicide attempts than those without ADHD and DBD, respectively. Maternal incarceration (*b* = 1.25, *SE* = 0.48, *p* = 0.01) was also significantly associated with number of suicide attempts, such that individuals with a currently incarcerated mother were expected to make on average 1.04 (*SE* = 0.62) suicide attempts whereas youth without a currently incarcerated mother were expected to make 0.30 attempts (*SE* = 0.12; Cohen’s *d* = 0.31). In addition, the traumatic stress and race-ethnicity covariates were significantly associated with number of suicide attempts (see Table 3).

A parallel model tested paternal incarceration as a predictor: although individuals with DBD were predicted to make significantly more suicide attempts (*b* = 0.69, *SE* = 0.29, *p* = 0.02) relative to those without DBD, paternal incarceration was unrelated to the number of suicide attempts (*b* = 0.15, *SE* = 0.36, *p* = 0.67). Traumatic stress and race-ethnicity remained significantly associated with number of suicide attempts (see Table 3).

## Discussion

Commercially sexually exploited (CSE) adolescent females are vulnerable to engaging in suicidal behaviors; until specific risk factors for these potentially fatal behaviors are identified, innovations in intervention and prevention will be delayed. This study analyzed the independent associations of ADHD, DBD, CD, and parental incarceration with the number of suicide attempts in an ethnoracially diverse sample of 360 girls with CSE histories. Controlling for age, race-ethnicity, and traumatic stress, positive ADHD and DBD (including oppositional defiant disorder) diagnoses prior to STAR Court involvement independently and positively predicted number of suicide attempts. In parallel models, maternal incarceration (not paternal) was specifically associated with a higher number of suicide attempts. As expected, traumatic stress was correlated with more suicide attempts; across all models, Latinx/Hispanic girls made more attempts than Black/African American girls. Finally, and consistent with previous reports of suicide attempts for Black (15.2%) and Latinx (11.9%) female adolescents [8], 14.7% of the sample endorsed a lifetime history of a suicide attempt.

ADHD and DBD diagnostic status as independent correlates of suicide attempts is consistent with past evidence that broad externalizing problems are associated with suicidality, particularly among girls. For example, in a 16-year prospective follow up of ethnoracially diverse girls, childhood ADHD symptoms robustly predicted suicide attempts [36] whereas externalizing problems (e.g., delinquency) were cross-sectionally and longitudinally positively correlated with suicide risk [39–41]. However, the mechanisms through which ADHD and DBD diagnoses increase risk for suicide attempts (and other suicidal thoughts and behaviors) remain unclear. One theory posits that it may be deficits in executive functioning capacity (i.e., self-regulatory cognitive processes including planning, inhibition, organization, set shifting, working memory, and problem solving) that link ADHD and DBD to suicidal outcomes [36]. Alternatively, the diathesis-stress model may be applicable whereby predisposed cognitive vulnerabilities (i.e., ADHD and DBD symptoms including impaired decision making) interact with early life adversity (i.e., CSE history) to increase risk of suicidality [55]. As such, future work is required to uncover the mechanistic pathways between ADHD and DBD diagnosis and eventual suicidal outcomes. Similarly, structural and socioemotional correlates of CSE (i.e., involvement in the juvenile legal, foster care, and child welfare system, living in high crime neighborhoods, elevated rates of physical health conditions) may compound risk for later suicidality, and thus warrant further examination.

Few, if any, previous studies have examined associations from specific dimensions of psychopathology, including specific diagnostic taxon (i.e., DBD/oppositional defiant disorder and CD), in a high-risk sample of ethnoracially diverse girls. Thus, these preliminary results suggest that improving suicidality outcomes among girls affected by CSE must assess and target lifetime histories of ADHD and externalizing problems. However, much of the literature on treatment for externalizing problems is focused on parenting interventions [56–60]. Considering that nearly 18% of adolescents in this sample had a parent currently incarcerated, many parents of adolescents impacted by CSE are unable to participate in these interventions. Thus, when specifically considering youth with CSE histories, including those involved in child welfare (e.g., residential/group homes), carceral settings, or other settings with familial instability (e.g., homeless), parenting- or family-focused interventions for these problems may be prohibitive [61, 62]. Thus, community and school-based interventions for externalizing problems must consider the family environment for high-risk adolescents vulnerable to suicidality and be modified to accommodate alternative caregivers for youth. Indeed, preliminary work has demonstrated a significant reduction in suicide in a group of adolescents enrolled in the Youth-Nominated Support Team Intervention for Suicidal Adolescents—Version II compared to those not enrolled [63]. This unique intervention capitalizes on support from youth-nominated “caring adults” (i.e., non-parental/guardian figures from their family, school, and community) provided to youth after psychiatric hospitalization for suicidal ideation or attempt [63]. In addition, a recent study of school-based mental health services in elementary-aged children found that interventions implemented by school personnel targeting externalizing problems showed particularly strong effects [64], attesting to the potential benefits of school-based interventions for at-risk youth. Similarly, when teachers identify and encourage parents to seek treatment for their children, this increases help seeking behavior for child externalizing disorders [65]. Thus, greater investment in school- and community-based mental health services may improve identification of problems and facilitate treatment delivery, particularly for vulnerable youth (e.g., ethnoracially diverse girls with CSE histories).

Although diagnostic status for ADHD and DBD predicted suicidality in this sample, it is also crucial to consider potential diagnostic biases secondary to race/ethnicity. Compared to non-Latinx White youth, Black and Latinx youth with learning and other mental health problems (i.e., trauma) were more likely to be misdiagnosed with oppositional defiant disorder and CD while simultaneously being less likely to be diagnosed with ADHD [66–72]. Indeed, these diagnostic disparities remain even with control of potentially related correlates (e.g., adverse childhood experiences, prior juvenile legal system involvement; [66, 68, 72]). Crucially, these disparities have serious clinical implications: for example, in the absence of an ADHD diagnosis, minoritized adolescents have less access to supportive services (i.e., therapy, medication), thus exacerbating health, educational, and juvenile legal disparities [69]. We therefore caution that unmeasured diagnostic biases, including in this study, may complicate strong inferences (especially in the context of broader externalizing problems).

Results from the current study also suggest the primacy of maternal incarceration, relative to paternal incarceration, with respect to the number of suicide attempts in youth. This suggests that the specific parent being incarcerated may differentially affect the development of youth. Considering 92% of parents in state and federal prisons are fathers [73], paternal incarceration may be relatively normalized in specific communities. Further, compared to children with incarcerated fathers, children with incarcerated mothers were more likely to have multiple risk factors relevant to the present study (e.g., mental health problems, sexual abuse) [74]. In particular, children with incarcerated mothers were more likely to be placed in foster care and other non-familial care situations compared to children with incarcerated fathers [75], suggesting that maternal incarceration may be more consequential to youth development and thus may result in a number of cumulative factors that increase risk for suicidal outcomes. As mentioned previously, recently, maternal incarceration, but *not* paternal incarceration, was positively correlated with creating a suicide plan among African American/Black adolescents [49]; this pattern is similar to those observed in the present study. Thus, adolescents with an incarcerated mother (vs. incarcerated father) may be at even greater compounded risk for detrimental life outcomes, including suicide; this constellation of risk factors may demarcate a subgroup of youth particularly vulnerable to suicidality. As such, future policies must provide for additional support and care for children of incarcerated parents, including mental health care and additional community support. Although there are multiple mentoring interventions for youth with incarcerated parents [76], these preliminary findings suggest that these interventions must integrate suicide prevention strategies to protect vulnerable youth from severe negative outcomes (i.e., suicide).

Despite not being the focus of the present study, trauma history was found to be a robust predictor of number of suicide attempts. This finding is in line with replicated evidence that traumatic experiences increase risk for suicide attempts. Indeed, one meta-analysis strove to identify which types of childhood trauma are most consequential for later suicide risk and found that history of sexual abuse was one of the most robust predictors of later suicide attempts [77]. Of course, CSE is considered a type of sexual abuse, and so it is understandable that much of the present population endorsed experiencing traumatic stress. Childhood traumas, including sexual abuse, also have an adverse impact on mental health broadly and on externalizing behaviors specifically [77]. Thus, experiencing childhood trauma may lead to engagement in externalizing behaviors consistent with ADHD and DBD diagnoses, resulting in cumulative risk for suicidality later in life. As such, it is critical that trauma be a target in future prevention and intervention strategies in order to reduce suicide risk.

### Limitations

The present study utilized a uniquely at-risk population of adolescent girls with CSE histories to test the concurrent associations of ADHD, DBD, CD, and parental incarceration with number of lifetime suicide attempts. We emphasize key study limitations. First, the structure and methodology of data collected by the STAR Court employed a temporally broad period to assess clinical correlates (i.e., diagnosis prior to STAR Court involvement) and frequently relied on dichotomous variables. Second, the clinical assessment precluded dimensional inferences (e.g., inattention vs. hyperactivity) that may differentially relate to suicidal behaviors. For example, children with the combined and hyperactive-impulsive subtypes of ADHD were at greater risk of making a suicide attempt compared to controls; the inattentive subtype was not [78]. Indeed, symptom dimensions may be particularly relevant given that inattention is more central to ADHD presentation in girls than in boys [79]. Third, given these cross-sectional data were limited by the absence of temporal features of externalizing problems (e.g., preceded or followed participant suicide attempts), directional inferences cannot be reliably made. Thus, future studies must utilize longitudinal designs to track the temporally-ordered relations among key constructs (e.g., ADHD, suicidality). Further, parental incarceration was limited to parents who were incarcerated at the time of STAR court entry, thus limiting more refined distinctions (e.g., length of incarceration). Finally, a structured clinical interview may have strengthened the assessment of suicide and suicidal behaviors. Instead, the study relied solely on retrospective youth self-report of number of suicide attempts, although this remains an evidence-based assessment method [80].

Despite these limitations, the present study provides novel, preliminary insight into potential correlates of suicide among ethnoracially diverse girls affected by CSE, a drastically understudied and at-risk population. ADHD and DBD, as well as maternal incarceration, were particularly salient correlates of number of suicide attempts. Future intervention strategies as well as policy development and implementation must strongly consider clinical correlates and family risk factors to prevent the onset and severity of suicidal behaviors.

## Summary

In summary, CSE is a significant public health concern that disproportionately victimizes ethnoracially minoritized girls and predicts severe mental health consequences, including suicide attempts. This study revealed specific clinical and familial correlates of suicide attempts amongst this at-risk population, including diagnosis of ADHD, diagnosis of DBD, and maternal incarceration. The implications of our findings suggest the importance of screening and assessment of these factors when developing and implementing interventions for suicidality among girls impacted by CSE. Indeed, until specific risk factors for suicidality are reliably identified, innovations in intervention and prevention will be severely delayed. As such, future intervention strategies as well as policy development and implementation must strongly consider clinical correlates and family risk factors to prevent the onset and severity of suicidal behaviors.

## Data Availability

Due to the sensitive nature of the data used in this manuscript, these data are not publicly available.
